# Comparison of Antioxidant Activities of Melanin Fractions from Chestnut Shell

**DOI:** 10.3390/molecules21040487

**Published:** 2016-04-22

**Authors:** Zeng-Yu Yao, Jian-Hua Qi

**Affiliations:** Key Laboratory for Forest Resources Conservation and Use in the Southwest Mountains of China, Ministry of Education, Southwest Forestry University, Kunming 650224, China; jhqi1977@163.com

**Keywords:** *Castanea mollissima*, colorant, free radical, pigment

## Abstract

Chestnut shell melanin can be used as a colorant and antioxidant, and fractionated into three fractions (Fr. 1, Fr. 2, and Fr. 3) with different physicochemical properties. Antioxidant activities of the fractions were comparatively evaluated for the first time. The fractions exhibited different antioxidative potential in different evaluation systems. Fr. 1, which is only soluble in alkaline water, had the strongest peroxidation inhibition and superoxide anion scavenging activity; Fr. 2, which is soluble in alkaline water and hydrophilic organic solvents but insoluble in neutral and acidic water, had the greatest power to chelate ferrous ions; and Fr. 3, which is soluble both in hydrophilic organic solvents and in water at any pH conditions, had the greatest hydroxyl (·OH) and 1,1-diphenyl-2-picryl-hydrazyl (DPPH·) radicals scavenging abilities, reducing power, and phenolic content. The pigment fractions were superior to butylated hydroxytolune (BHT) in ·OH and DPPH· scavenging and to ethylene diamine tetraacetic acid (EDTA) in the Fe^2+^–chelation. They were inferior to BHT in peroxidation inhibition and O_2_·^−^ scavenging and reducing power. However, BHT is a synthetic antioxidant and cannot play the colorant role. The melanin fractions might be used as effective biological antioxidant colorants.

## 1. Introduction

Reactive oxygen species (ROS), including hydroxyl radicals (·OH), superoxide anions (O_2_·^−^), hydrogen peroxide (H_2_O_2_), and singlet oxygen (^1^O_2_), are natural products of the various physiological processes, such as respiration chain, innate immune responses and the reduction of oxygen by the catalysis of enzymes (oxidases and oxygenases) [1]. Some exogenous factors, such as environmental pollutants, industrial chemicals, infectious agents, deep-fried foods, cigarette smoke, radiation, and some xenobiotics, may also produce ROS [2]. At moderate concentrations, ROS play an important role as regulatory mediators in signalling processes. At high concentrations, however, they are harmful to living organisms [1]. They cause oxidative damage to cell components, leading to cancer, aging, atherosclerosis, diabetes, neurodegenerative diseases, cataracts, and other health problems [3,4,5]. Although human beings have antioxidant defences and repair systems against the oxidative damage, these inherent activities can be inefficient. Increasing the intake of exogenous antioxidants—from the diet may help a living system to keep an adequate antioxidant status and normal physiological functions [6]. In addition, ROS are known to play an important role in food deterioration during processing and storage that leads to rancidity, off-flavor, textural degradation, nutritional value decrease, economic losses, and health risks. The addition of an antioxidant prolongs the shelf life of food products. For these reasons, an increasing number of investigations have been carried out to find new antioxidants.

Color is an important attribute of food acceptance by consumers. It is appreciated for the aesthetic value and plays a role for quality judgment. Therefore, colorants have been widely used in the food industry to improve appearance and acceptability of the products for consumers.

Melanins are pigments with high molecular weight synthesized by organisms in all living kingdoms [7,8]. Their structures are complex and varied by the combination of monomeric units and the environmental conditions during polymerization. Extraction and purification of melanins usually involves harsh chemical treatments, which can change the natural state of the polymers. The complex nature and the exceptive isolation make them difficult to characterize, and their molecular structures have not yet been fully identified. Chemical analyses and spectroscopic methods including ultraviolet–visible (UV–VIS), Fourier transform infrared (FT–IR), electron spin resonance (ESR), and solid-state nuclear magnetic resonance (NMR) are usually used to reveal structural information of the polymers [9]. Recent research on natural bioactive colorants and antioxidants as multifunctional additives has increased because of consumer interest in nutritional and healthy food [10]. Some natural melanins have been deemed as potential healthy food additives that act as both colorants and antioxidants. The multiple antioxidant activities of melanins were discovered [10,11], as well as radioprotection [12], anti-tumor [13], anti-HIV [14], immuno-stimulating [15], antivenin [16], and anti-inflammatory activity [17].

China is the world’s largest chestnut producer, with about 1.65 million tons in 2013 [18]. Chestnut shells are a waste of the food industry. This byproduct contains about 15% brown pigment which has been identified as an herbal melanin and developed as a food colorant with antioxidant properties [19,20,21]. However, the crude pigment from the chestnut shells was partly dissolved in neutral and acidic aqueous solutions and hydrophilic organic solvents. Hence, it is hardly used in these solvents. In the previous study [21], we developed a technique to fractionate the pigment into three mixtures of ingredients (Fr. 1, Fr. 2, and Fr. 3) according to the solubility in various solvents. This provided an opportunity to choose an appropriate fraction for use according to the solubility needs, thus application scopes of the pigment can be expanded. We have intensively characterized physicochemical properties of the pigment fractions by chemical analyses, UV–VIS, FT–IR, ESR, and ^13^C-NMR, and found they were different in chemical structures and properties [21,22]. Fr. 1 is soluble in alkaline water (above pH 10), but insoluble in neutral and acidic water and all the tested organic solvents (methanol, ethanol, acetone, acetic ether, chloroform, ethyl ether, petroleum ether, and hexane). Fraction 2 is soluble in alkaline water and hydrophilic organic solvents (methanol, ethanol, and acetone), but insoluble in neutral and acidic water and lipophilic organic solvents (acetic ether, chloroform, ethyl ether, petroleum ether, and hexane). Fraction 3 is soluble in the water regardless of the pH and the hydrophilic organic solvents, but insoluble in the lipophilic organic solvents. The pigment-bound proteins and polysaccharides are the most abundant in Fr. 1 while Fr. 2 has the highest aromaticity. The stabilities of the pigment fractions were also reported in our previous study [22]. All the fractions are stable to reductants, sucrose, Na^+^, and Mg^2+^ but unstable to Al^3+^, Ca^2+^, Fe^2+^, Fe^3+^, Cu^2+^, and Zn^2+^. Fraction 1 is relatively stable to heating and UV light while Fr. 3 is the most sensitive.

In this study, we comparatively evaluated their antioxidant activities employing various *in vitro* assay systems to provide experimental evidence for their antioxidant activity and potential for further development and utilization.

## 2. Results and Discussion

### 2.1. Inhibition of Lipid Peroxidation

**Figure 1:**
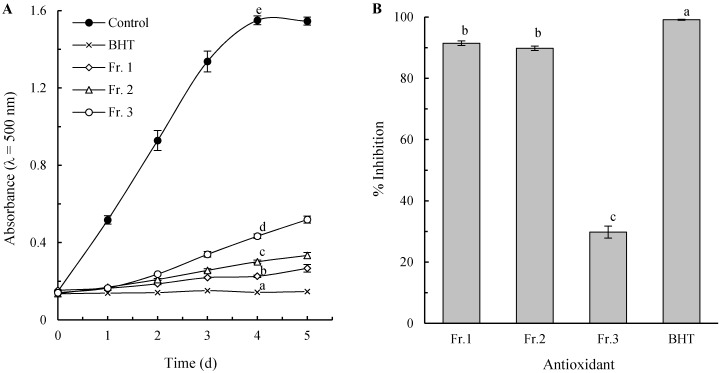
Peroxidation of linoleic acid and the inhibition effects of pigment fractions from chestnut shell evaluated by FTC (**A**) and TBA (**B**) methods. The lower case letters above the curves and bars indicate significant differences between means (*p* < 0.05).

Lipid peroxidation has been researched intensively for its role in the alteration of foods and in the oxidation by ROS [23,24]. In this study, both of FTC and TBA methods were used to measure the inhibiting activities against lipid peroxidation of linoleic acid. The FTC method determines the amount of peroxide (such as lipid hydroperoxide) produced at the initial stage of lipid oxidation, and a lower absorbance indicates a higher antioxidant activity. Figure 1A shows the changes in the absorbance for each pigment fraction during 5 days of incubation at 37 °C, in comparison with BHT and control. The absorbance for all the treatments (except the control) increased in proportion to the incubation time. All the pigment fractions and BHT showed a significantly lower increment in the rate compared with the control (*p* < 0.05), indicating their strong lipid peroxidation inhibiting. It is also easy to verify by the absorbance after 4 d incubation, when the data obviously show the inhibiting efficiency decrease in the order BHT > Fr. 1 > Fr. 2 > Fr. 3. During the oxidation process, peroxides are gradually decomposed to lower molecular weight compounds, among which malondialdehyde (MDA) is the most important derivative and considered to be a useful biomarker to look into the final stage of lipid peroxidation. The amount of MDA can be measured by the TBA method. As shown in Figure 1B, the antioxidant activity based on TBA method was similar to that on FTC method, except there was no significant difference between Fr. 1 and Fr. 2 (*p* > 0.05).

### 2.2. Hydroxyl Radical Scavenging Activity

**Figure 2:**
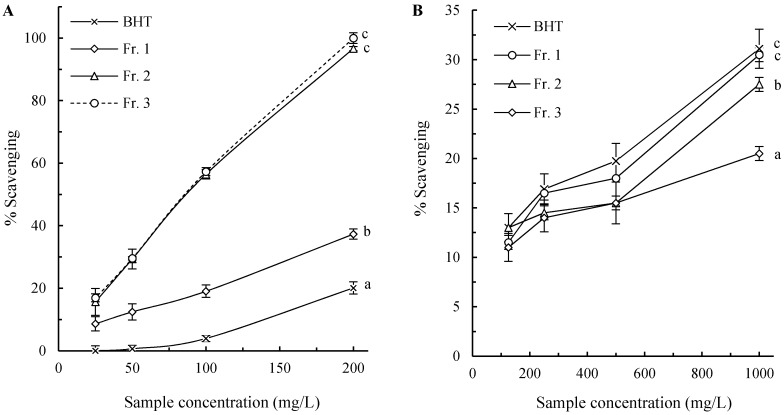
Dose dependent hydroxyl radical (**A**) and superoxide anion radical (**B**) scavenging effects of pigment fractions from chestnut shell. The lower case letters on the right of the curves indicate significant differences between means at sample concentration of 200 and 1000 mg/L for hydroxyl radical superoxide and anion radical scavenging, respectively (*p* < 0.05).

The hydroxyl radicals are the most reactive ROS generated in the body, which react with almost all components within the cell, including lipids, proteins and nucleotides [23]. Due to their high toxicity, scavenging capacity towards these radicals is widely used as an important indicator to evaluate antioxidants [25]. The effect of the pigment fractions on ·OH produced by the Fenton reaction was determined, and the results are shown in Figure 2A. All the tested samples had scavenging activities towards ·OH dose-dependently. The pigment fractions were more efficient than BHT at the tested concentrations (*p* < 0.05) with Fr. 2 and Fr. 3 having similar (*p* > 0.05) and higher activity than Fr. 1 (*p* < 0.05).

### 2.3. Superoxide Anion Radical Scavenging Activity

Superoxide radicals are the most common ROS *in vivo* and involve in many pathological processes [1]. They can give birth to stronger ROS such as singlet oxygen and hydroxyl radicals, and initiate peroxidation of lipids, despite their weak oxidizing capacities [23]. Thus, scavenging these radicals would be promising for food preservation, healthcare, and treatment of related diseases. In this study, the scavenging capacity of the melanin fractions from chestnut shells towards superoxide anion radicals was evaluated by using the pyrogallol autoxidation system. As shown in Figure 2B, the pigment fractions, as well as BHT, showed weak superoxide radical scavenging activities even at high concentration (1000 mg·L^−1^). The scavenging capacity of the fractions was Fr. 1 > Fr. 2 > Fr. 3 with BHT having insignificantly higher activity than Fr. 1 (*p* > 0.05).

### 2.4. DPPH· Scavenging Activity

**Figure 3:**
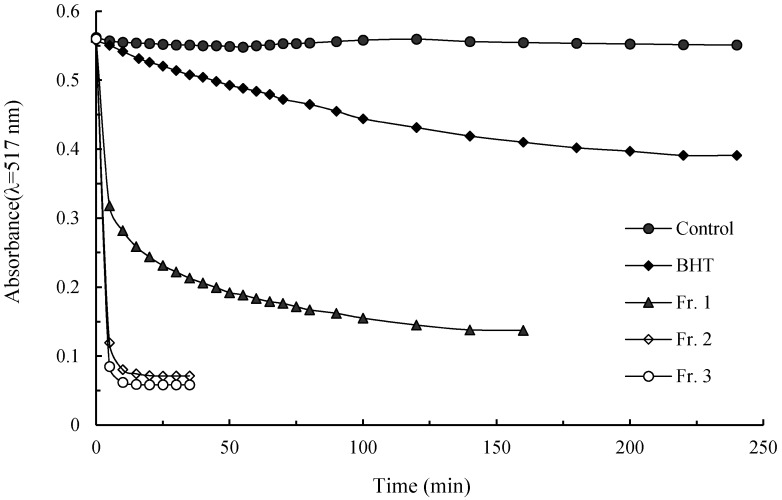
Contact time dependent DPPH· scavenging effects of pigment fractions from chestnut shell.

DPPH· is a stable organic free radical, and scavenging of this radical is related to the inhibition of lipid peroxidation [24]. The ability to scavenge DPPH· is extensively used as an easy, rapid, and sensitive way to evaluate free radical-scavenging capacities of natural antioxidants [26]. This methodology is based on the theory that DPPH· is scavenged by an antioxidant through a donation of hydrogen to form a stable DPPH−H molecule. Antioxidants, according to their reaction kinetics with DPPH·, can be classified into three general groups: fast-kinetics, fast + slow-kinetics, and slow-kinetics. The fast-kinetics antioxidants have antiradical groups capable of fast hydrogen atom transfer but incapable of slow hydrogen atom transfer. The slow-kinetics ones have antiradical groups capable of slow hydrogen atom transfer but incapable of fast hydrogen atom transfer. The fast+slow-kinetics ones have antiradical groups capable of both fast and slow hydrogen atom transfer [26]. Figure 3 shows the typical kinetic behaviors of the pigment fractions and BHT as radical scavengers toward DPPH· at sample concentration of 500 mg·L^−^^1^. The DPPH· decay curve of BHT displays the classical slow-kinetics. Fr. 1 shows the fast + slow-kinetics with two consecutive stages. In this curve, the absorbance rapidly lessened from 0.56 to 0.32 in the first 5 min (fast kinetics) followed by a further but gradual decline from 0.32 to 0.14 during the next 135 min (slow-kinetics). Fractions 2 and 3 behaved as the typical group of fast-kinetics, and the absorbencies rapidly decreased and reached a steady state in 10 min. These results determined that Fr. 1 has antiradical groups capable of both fast and slow hydrogen atom transfer, and the antiradical capacities of Fr. 2 and Fr. 3 would mainly correspond to fast hydrogen atom transfer. 

The EC_50_ is a parameter widely used to measure the antiradical efficiency. The lower the EC_50_ is, the higher the antioxidant power will be. In the present study, all the pigment fractions have DPPH· scavenging activity. Among the three pigment fractions, Fr. 3 exhibited the strongest scavenging efficiency, and its EC_50_ value was 66.5 ± 1.0 mg·L^−1^, followed by Fr. 2 (EC_50_ = 75.5 ± 2.1 mg·L^−1^) and Fr. 1 (EC_50_ = 292.2 ± 3.9 mg·L^−1^). The activities of all the pigment fractions are greater than that of BHT (EC_50_ = 722.3 ± 4.4 mg·L^−1^), which is a synthetic antioxidant used in food industry.

### 2.5. Reducing Power

**Figure 4:**
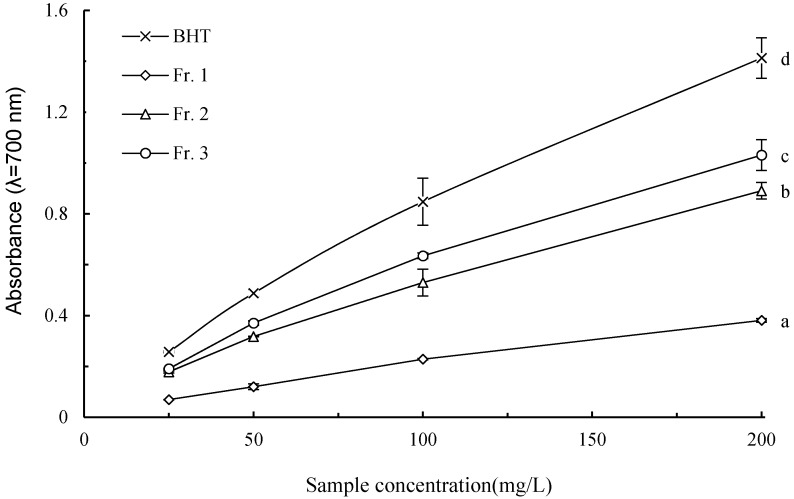
Dose dependent reducing power of pigment fractions from chestnut shells. The lower case letters on the right of the curves indicate significant differences between means at sample concentration of 200 mg/L (*p* < 0.05).

The reducing power of a compound could be used as an indicator of its potential antioxidant capacity, and the ability to reduce Fe^3+^ to Fe^2+^ is often assayed as an indicator of electron-donating activity [25,27]. As evidenced in Figure 4, all the pigment fractions, as well as BHT, presented linearly dose-dependent increases in absorbance, and their reducing power followed the order of BHT > Fr. 3 > Fr. 2 > Fr. 1.

### 2.6. Iron Chelating Ability

**Figure 5:**
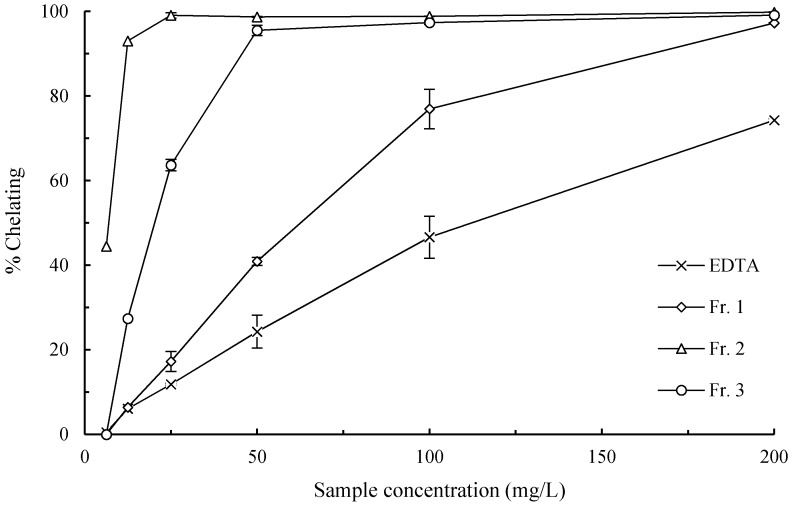
Dose-dependent metal chelating ability of pigment fractions from chestnut shell.

It has been recognized that transition metal ions play an important part in the ROS generation *in vivo*. Iron can stimulate lipid peroxidation by catalyzing the Haber–Weiss reaction and catalyze lipid hydroperoxides to form chain-carrying peroxyl and alkoxyl radicals which increase the rate of chain reinitiation or propagation [28]. Chelating agents can modulate the catalytic activity of the metal and serve as secondary antioxidants [29]. Therefore, the chelating ability is an important indicator of the antioxidative property of a compound. EDTA, a strong metal chelator, was used as the standard metal chelator in this study. As revealed in Figure 5, chestnut shell melanin fractions demonstrate excellent capacities for iron binding. Their Fe^2+^–chelating efficiencies, expressed by EDTA equivalent were 1762 ± 49, 16778 ± 588 and 5666 ± 304 mg EDTA g^−1^ pigment for Fr. 1, Fr. 2, and Fr. 3, respectively. This suggested that their peroxidation inhibitory ability may be associated with their iron binding capacity.

### 2.7. Phenolic Concentration

Melanins can be at various degrees of oxidation, and their antioxidant activity depends on their oxidative state. Phenolic groups in melanins were turned into quinone groups during the oxidation, diminishing the antioxidative capacity of the melanins [11]. Phenolic content is an indicator to evaluate the oxidative degree of melanins. In addition, many studies have revealed the phenolic contents in some phyto-product are related to their antioxidant activities [30]. The antioxidant profiles of phenolic groups attribute to their multiple capacities, including chelating transitional metals, reducing oxygen from the singlet state to the triplet one, removing radicals, and inactivating oxidases, such as the xanthine oxidase. In this study, the total amounts of phenolic groups in the fractions of the chestnut shell pigment were determined by the Folin-Ciocalteu method, and gave the following order: Fr. 3 (498.8 ± 7.5 mg·GAE·g^−1^) > Fr. 2 (449.5 ± 4.5 mg·GAE·g^−1^) > Fr. 1 (207.2 ± 1.5 mg·GAE·g^−1^). This order is same as the aforementioned results in ·OH and DPPH· scavenging capacity and reducing power, suggesting phenolic groups could be playing some role in these respects. 

There are few reports on the relationship between the antioxidant activity and chemical structure of melanin [11,31]. Regression analysis established that melanin fractions from *Inonotus obliquus* with a high content of pyrocatechols and O-containing functional groups (O/C ratio) and degree of aromaticity (H/C ratio) showed high antioxidant activity. These results were not corroborated in this study, however the difference may be caused by proteins and polysaccharides bound on the melanin fractions from chestnut shells. The strongest inhibiting efficiency of lipid peroxidation and scavenging activity of superoxide radicals by Fr. 1 could possibly be explained by proteins and polysaccharides in this pigment fraction that acted as free-radical traps [22]. The total acidic groups, including carboxyl and phenolic hydroxyl, followed the order of Fr. 2 > Fr. 3 > Fr. 1 [32], which is the same as the order for iron chelating ability. It was reported that the carboxylic, hydroxyl, and amine groups are potential binding sites for metal ions in melanins [33,34,35], and may provide the chelating power of the melanin fractions.

## 3. Materials and Methods 

### 3.1. Chemicals

1,1-diphenyl-2-picryl-hydrazyl (DPPH·) and 3-(2-pyridyl)-5,6-bis (4-phenyl-sulfonic acid)-1,2,4-triazine (Ferrozine) were purchased from Alfa Aesar (Karlsruhe, Germany). Linoleic acid was obtained from Sigma-Aldrich (Sternheim, Germany). All other reagents used were analytical grade commercially available. Pyrogallol was purified by sublimation prior to use.

### 3.2. Preparation of Melanin Fractions

The melanin fractions (Fr. 1, Fr. 2, and Fr. 3) were obtained from our previous work [21]. The crude extract was obtained by maceration of 60 g chestnut (*Castanea mollissima*) shell in a 900 mL solution of 0.5 mol·L^−1^ NaOH for 36 h at 40 °C. The fresh extract was centrifuged, and the supernatant was subjected to the Sevag method to remove free proteins. The fractionation protocol involved the following steps:

Step (1): The solution was acidified to pH 2. After incubation for 12 h, it was separated into sediment and supernatant by centrifugation. The former was re-dissolved in basic water (pH 11), and the insoluble was removed by the centrifugation. The basic supernatant was re-acidified and centrifuged. The reprecipitation was carried out several times until the acidic supernatant was nearly colorless.

Step (2): The precipitate was washed with ethanol and separated from the liquid by centrifuge. The wash was continued while the ethanol was colored.

Step (3): The residue was further washed three times with ethyl acetate and then three times with acetone and finally dried at 60 °C. This fraction was Fr. 1.

Step (4): The ethanol supernatants in step (2) were collected and two volumes of petroleum ether were added to precipitate the pigment in the ethanol. After centrifugation, the sediment was re-dissolved in ethanol and re-precipitated by petroleum ether three times, then dried at 60 °C. This fraction was Fr. 2.

Step (5): All the acidic supernatants in step (1) were collected and passed through an open column packed with the Amberlite XAD-8 resin. The column was washed with three column volumes of water of pH 2 (adjusted by HCl) and subsequently rinsed with distilled water until a silver nitrate test for chloride ions was negative. The pigment was eluted with ethanol, precipitated by adding petroleum ether, and dried. This fraction was Fr. 3.

To evaluate antioxidant activities, stock solutions of the pigment fractions were prepared by dissolving 100 mg of each sample in 80 mL of 0.2% NH_4_OH under nitrogen atmosphere at 40 °C on a shaker for 24 h. After removing the ammonia in a rotary evaporator, the solutions were diluted to 100 mL with distilled water. Working solutions were prepared by a suitable dilution of the stock solution with distilled water.

### 3.3. Ferric Thiocyanate (FTC) Assay

The method of Kikuzaki and Nakatani [36] was slightly modified. A glass-stoppered test tube containing a mixture of 0.5 mL of 100 mg·L^−1^ sample solution, 2 mL of 25 g·L^−1^ linoleic acid (in 99.5% ethanol), and 8 mL of 50 mmol·L^−1^ phosphate buffer at pH 7.0 was placed in a water bath at 37 °C in the dark. To 0.1 mL of this reaction mixture at 24 h intervals, 9.7 mL of 75% ethanol, and 0.1 mL of 300 g·L^−1^ ammonium thiocyanate were added. Precisely 3 min after addition of 0.1 mL of 20 mmol·L^−1^ ferrous chloride in 35 g·L^−1^ hydrochloric acid to the reaction mixture, the absorbance was measured at 500 nm on a UV−2102 PCS spectrometer (UNICO, Shanghai, China). This step was repeated every 24 h until one day after the control reached its maximum absorbance value. The synthetic antioxidant butylated hydroxytolune (BHT) was used as a positive control.

### 3.4. Thiobarbituric Acid (TBA) Assay

The reaction mixtures from the final incubation day of the FTC method were the initial solutions for TBA assay. A centrifuge tube containing 1 mL of the reaction mixture, 2 mL of 100 g·L^−1^ aqueous trichloroacetic acid (TCA), and 2 mL of 8 g·L^−1^ aqueous TBA was placed into a boiling-water bath for 10 min. After cooling, it was centrifuged at 3000 rpm for 30 min. Antioxidant activity was based on the absorbance of the supernatant at 532 nm. The percent inhibition of linoleic acid peroxidation by each pigment fraction was calculated by the following equation:

% Inhibition = (*A*_0_ − *A*_1_)/*A*_0_ × 100 (1)

where *A*_0_ and *A*_1_ are the absorbances of the control (without sample) and the experimental (with sample) reactions, respectively.

### 3.5. Hydroxyl Radical Scavenging Assay

The method of Ling [25] was slightly modified. One milliliter of 0.1 g·L^−1^ H_2_O_2_ was added in a test tube that contained a mixed solution of 1 ml orthophenanthroline (0.75 mmol·L^−1^), 2 mL phosphate buffer (PBS, 0.2 mol·L^−1^, pH 7.4), 1 mL FeSO_4_ (0.75 mmol·L^−1^), and 1 mL sample of different concentrations (25–200 mg·L^−1^). The test tube was incubated in a water bath at 37 °C for 1 h, after which the absorbance (*A_s_*) was measured at 536 nm. In the negative control group, the samples were replaced by 1mL distilled water, and their absorbance was denoted as *A_n_*. In the positive control group, H_2_O_2_ was replaced by 1 mL distilled water, and their absorbance was denoted as *A_p_*. The percent scavenging of hydroxyl radical was expressed by the following equation:

% Scavenging = (*A_s_* − *A_p_*)/(*A_n_* − *A_p_*) × 100 (2)

### 3.6. Superoxide Radical-Scavenging Assay

The superoxide radical scavenging capacity was assayed by using a modified pyrogallol autoxidation system [37]. The reaction mixture was prepared in a cuvette containing 3.0 mL Tris–HCl Buffer (50 mmol·L^−1^, pH 8.2) and 0.5 mL sample of different concentrations (100–1000 mg·L^−1^). The cuvette was incubated at 25 °C for 10 min, and then 200 μL of 10 mmol·L^−1^ pyrogallic acid (prepared in 10 mmol·L^−1^ HCl) was added. The absorbance of the reaction mixture at 420 nm was recorded immediately at 30 s intervals. The auto-oxidation rate constant (*K_b_*) of pyrogallic acid was obtained from the slope on the plot of the absorbance at 420 nm against time. Distilled water instead of the sample was served as the negative control. The inhibition efficiency was calculated by the following equation:

% Scavenging = (*K_b,control_* − *K_b,sample_*)/*K_b,control_* × 100 (3)

### 3.7. DPPH Radical Scavenging Assay

The DPPH· scavenging activity was assayed by a slightly modified method of Sendra *et al.* [26] A DPPH· solution of 0.1 mmol·L^−1^ in methanol was prepared and left in a refrigerator for 2 h in order to stabilize. Five millilitres of this solution was mixed with 0.1 mL of the sample. The reaction mixture was incubated in the dark at room temperature, and the absorbance at 515 nm was recorded at different time points. The decreasing absorbance of the DPPH· solution indicated an increase of the DPPH radical-scavenging activity. The percent radical scavenging activity was calculated by the following equation:

% Scavenging = [*A_control_* − (*A_sample_* − *A_blank_*)]/*A_control_* × 100 (4)

Methanol (5 mL) plus each sample solution (0.1 mL) was used as a blank. DPPH· solution (5 mL) plus distilled water (0.1 mL) was used as a negative control.

The EC_50_ value, defined as the concentration of the test sample necessary to inhibit 50% of the scavenging activity, was calculated from the linear regression of the radical-scavenging percentage *versus* the logarithm of the sample concentration.

### 3.8. Reducing Power Assay

The reducing power was quantified according to the method of Oyaizu [27]. In brief, one milliliter of the sample solution (25–200 mg·L^−1^) was mixed with 2.5 mL of phosphate buffer (0.2 mol·L^−1^, pH 6.6) and 2.5 mL of 10 g·L^−1^ potassium ferricyanide. The mixture was incubated at 50 °C for 20 min. Then 2.5 mL of 100 g·L^−1^ TCA solution was added to the reaction mixture, and the mixture was centrifuged at 5000 rpm for 10 min. The supernatant (2.5 mL) was mixed with 2.5 mL of distilled water and 0.5 mL of 1 g·L^−1^ ferric chloride. After a 10 min reaction time, the absorbance of the resulting solution was measured at 700 nm. Increased absorbance of the reaction mixture indicated increased reducing power.

### 3.9. Ferrous Ion-Chelating Assay

The chelating activity for ferrous ions was measured according to the method of Dinis *et al.* [29] with slight modifications. Briefly, two milliliters of different concentrations (6.25–200 mg·L^−1^) of the sample were mixed with 0.2 mL of 2 mmol·L^−1^ FeCl_2_. After a five-minute reaction time, 0.4 mL of 5 mmol·L^−1^ ferrozine was added into the mixture followed by vigorous shaking. After standing at room temperature for 10 min, the absorbance at 562 nm was measured. The negative control was prepared without the test compound. Ethylene diamine tetraacetic acid (EDTA) served as a positive control. The percent chelating activity of the test compound on Fe^2+^ was calculated by the following equation.

% Chelating activity = (*A*_0_ − *A*_1_)/*A*_0_ × 100 (5)

where *A*_0_ and *A*_1_ are the absorbances of the control (without sample) and the experimental (with sample) reactions, respectively.

### 3.10. Determination of Total Phenol Groups

The assay solutions of the melanin fractions were prepared according to the procedure designed for melanin from tea leaves [15]. Total phenol groups in the pigment fractions were assayed quantitatively according to the Folin-Ciocalteu procedure [38], with some slight modifications. Folin–Ciocalteu reagent (2.5 mL) with a concentration of 0.2 mol·L^−1^ was added to 0.5 mL of each 50 mg·L^−1^ samples. After an interval of 5 min, two milliliters of 75 g·L^−1^ sodium carbonate was added. After a 2 h incubation at room temperature, the absorbance at 760 nm was read against a mixture of water and reagents. The concentration of phenol groups was expressed as milligrams of gallic acid equivalent (GAE) per gram dry material.

### 3.11. Statistical Analysis

All the experiments were carried out in triplicate, and the results were shown as mean ± standard deviation. Statistical analysis was conducted using one-way ANOVA followed by LSD multiple-range test using the computer software SPSS 21.0 (SPSS Inc., Chicago, IL, USA) for Windows. Statistical significance was defined as *p* < 0.05.

## 4. Conclusions 

This study is the first to report the antioxidant activities of the melanin fractions from chestnut shells. The pigment fractions exhibited different antioxidative potential in the different evaluation system. The orders were Fr. 1 > Fr. 2 > Fr. 3 for peroxidation inhibition and O_2_·^−^ scavenging, Fr. 2 > Fr. 3 > Fr. 1 for Fe^2+^–chelation, and Fr. 3 > Fr. 2 > Fr. 1 for OH and DPPH·scavenging, reducing power and phenolic content. The pigment fractions were superior to BHT in ·OH and DPPH· scavenging and to EDTA in the Fe^2+^–chelation. All fractions were inferior to BHT in peroxidation inhibition and O_2_·^−^ scavenging and reducing power. However, BHT is a synthetic colorless substance that solely acts as an antioxidant without colorant function. Based on the discussion above, the pigment fractions are effective antioxidative colorants.
